# Extended network analysis: from psychopathology to chronic illness

**DOI:** 10.1186/s12888-021-03128-y

**Published:** 2021-02-27

**Authors:** Adela-Maria Isvoranu, Edimansyah Abdin, Siow Ann Chong, Janhavi Vaingankar, Denny Borsboom, Mythily Subramaniam

**Affiliations:** 1 Department of Psychology, Psychological Methods, University of Amsterdam, Nieuwe Achtergracht 129B, 1018 WT Amsterdam, The Netherlands; 2 Research Division, Institute of Mental Health, Singapore, Singapore

**Keywords:** Mental health, Physical health, Chronic illness, Psychopathology, Network psychometrics, Network analysis, Functioning

## Abstract

**Background:**

Understanding complex associations between psychopathology and chronic illness is instrumental in facilitating both research and treatment progress. The current study is the first and only network-based study to provide such an encompassing view of unique associations between a multitude of mental and physical health-related domains.

**Methods:**

The current analyses were based on the Singapore Mental Health Study, a cross-sectional study of adult Singapore residents. The study sample consisted of 6616 respondents, of which 49.8% were male and 50.2% female. A network structure was constructed to examine associations between psychopathology, alcohol use, gambling, major chronic conditions, and functioning.

**Results:**

The network structure identified what we have labeled a *Cartesian graph*: a network visibly split into a psychopathological domain and a physical health domain. The borders between these domains were fuzzy and bridged by various cross-domain associations, with functioning items playing an important role in bridging chronic conditions to psychopathology.

**Conclusions:**

Current results deliver a comprehensive overview of the complex relation between psychopathology, functioning, and chronic illness, highlighting potential pathways to comorbidity.

**Supplementary Information:**

The online version contains supplementary material available at 10.1186/s12888-021-03128-y.

## Background

Mental illness is one of the most pressing contemporary problems, with impact on health, social and economic issues. Despite significant research efforts, common mental disorders within the general population remain a major concern, with reports as high as 28.8% for anxiety disorders, 20.8% for mood disorders, and 14.6% for substance use disorders [1], as well as rates of up to 40% for subjects with a mental disorder to meet criteria for another class of lifetime disorder [2].

In addition to high comorbidity between mental disorders, there is also vast evidence that people with common mental health conditions are at higher risk of developing physical illness, and conversely people with a diagnosis of physical illness are at higher risk of developing mental health conditions [3]. For instance, robust associations between immunological/ inflammatory conditions and mood disorders [4] have been identified, with depressed patients being 60% more likely to develop diabetes than their non-depressed counterparts and prevalence rates of diabetes as high as three times greater in subjects with bipolar disorder [3]. Further, in patients with schizophrenia, cardiovascular disease is the most common cause of death [5]. Of note, while highly relevant, the comorbidity between mental health and physical conditions is often neglected [6]. Here we argue better understanding this comorbidity may lead to improved prognosis and outcomes. The aim of the current study was therefore to delve into the relationship between mental and physical health conditions, as to highlight features important in explaining the development of this comorbidity.

In recent years it has been suggested that some symptoms of particular diagnoses, but not all, may account for the comorbidity patterns between diagnoses, indicating that symptoms may have a unique role and may not be interchangeable [7]. This line of reasoning, now known as the *network framework* [8] has been proposed as an innovative tool in the study of psychopathology, and in the past decade it has grown prominent in the fields of psychiatry and clinical psychology [9]. Within this framework, the focus shifts from the diagnostic level to the symptom level, with the aim to highlight the unique role of symptoms, and their potential causal associations. Network structures may therefore be useful tools to study both within-diagnoses and between-diagnoses symptom associations.

Further, the network approach suggests that the boundaries between mental and physical disorders are porous [10], as physical symptoms can cause psychopathological symptoms (e.g., pain - > fatigue - > depressed mood) and vice versa (e.g., depressed mood - > alcohol use - > liver damage). If so, it is crucial to chart the pathways by which these influence each other, as to ultimately reach better treatment targets. The current research aims to highlight features that may account for comorbidity between diagnoses and provide an encompassing view of unique associations between psychopathological conditions and chronic illness and functioning. To this end, we aimed to constructed a large network structure, encompassing a multitude of symptoms and other health-related dimensions, ranging from general psychopathology, to psychosis, alcohol use, chronic physical conditions and functioning and health-related quality of life (HRQoL). To our knowledge, this is the first and only network-based study encompassing such as multitude of health-related domains, as well as the only existing network study concerned with the comorbidity between mental and physical health conditions.

## Methods

### Sample

The sample analyzed (*n* = 6616 respondents) was part of the Singapore Mental Health Study (SMHS), a cross-sectional, population-based, epidemiological study of adult Singapore residents aged 18 years and above. The study aimed to establish lifetime and 12-month prevalence of mental disorders, as well as the current use of mental health services, treatment gaps and loss of role functioning. The subjects were randomly selected from a national registry that maintains the names, sociodemographic details (e.g., age, gender and ethnicity), and household addresses of all residents in Singapore. Inclusion criteria were being a Singapore citizen or resident, 18 years or older, and able to speak and understand English, Chinese or Malay. Exclusion criteria included being incapable of doing an interview due to severe physical or mental health conditions, language barriers, living outside the country, institutionalized or hospitalized throughout the duration of the survey period, as well as incomplete or incorrect addresses. A disproportionate stratified sampling was used where the 3 main ethnic groups (Chinese, Malays, and Indians) were sampled in equivalent proportion of about 30% each. Further details of the sample are available in the cited papers [11, 12].

### Measures

All measures used in this study are reported in Table 1 and described in Appendix 1 in the Supplement. Due to the skip-structure of the interviews, we selected and included only items that were answered by the full sample, focusing on sub-clinical levels of psychopathology. Overall, we included items pertaining to the World Health Organization-Composite International Diagnostic Interview (WMH-CIDI) [13], a modified CIDI checklist of chronic medical conditions, the South Oaks Gambling Screen (SOGS) [14], and the EQ-5D [15].

**Table 1:** Study measures

Domain	Instrument	Measure	Items
General Psychopathology	WMH-CIDI	Screening Section	26 items measuring: smoking, mental and physical health, anxiety, intermittent explosive disorder, depression, generalized anxiety attack, specific phobias, social phobia, agoraphobia, attention deficit hyperactivity disorder, oppositional defiant disorder, separation anxiety.
Psychosis	WMH-CIDI	Psychosis Screen	1 item measuring psychosis
Obsessive-Compulsive Disorder	WMH-CIDI	Obsessive-Compulsion Disorder Section	1 item measuring compulsions, 1 item measuring obsession
Alcohol Use	WMH-CIDI	Alcohol Use	1 item measuring age of first alcoholic beverage
Gambling	SOGS	Lifetime Gambling	1 item measuring lifetime gambling
Major Chronic Conditions	CIDI checklist of chronic medical conditions	Major Chronic Medical Conditions	6 items measuring presence of asthma, high blood sugar / diabetes, hypertension, back problems, migraine headaches, and other chronic conditions
Health-Related Quality of Life	EQ-5D	Quality of Life and Functioning	5 items measuring mobility, self-care, usual activities, pain or discomfort, and anxiety or depression.

Abbreviations: *WMH-CIDI* World Health Organization-Composite International Diagnostic Interview, *SOGS* South Oaks Gambling Screen

### Statistical analysis

#### Network construction

We constructed an undirected, weighted network model and included all measures described in Table 1 as nodes, with each edge in the network reflecting the pairwise conditional relation between two nodes, while controlling for all other nodes in the network. We fitted an Ising Model to the data using the eLasso technique implemented in the *IsingFit R*-package, version 0.3.1 [16]. The technique is based on the Ising Model as used in statistical physics, and uses l_1_ regularized logistic regression [17], commonly referred to as the e*Lasso*, conjointly with the extended Bayesian Information Criterion (EBIC) [18]. The method has been shown successful in identifying the most relevant features of a network constructed from binary data [16].

We visualized the network using the *qgraph R*-package version 1.6.4 [19]. Blue (red) edges represent positive (negative) associations, and the thicker the edge, the stronger the association between two nodes [20]. The layout of the network is based on the Fruchterman-Reingold algorithm [21], which places nodes with stronger and/or more connections closer to the center of the network and to each other.

#### Centrality analysis

To investigate the centrality of each node in the network, we computed *strength* [22] as a centrality measure. Node strength is a measure of the number and strength of connections, quantifying how well a node is directly connected to other nodes. Previous research showed strength to be the most robust centrality measure [23].

#### Network stability

To investigate the robustness and replicability of results we performed accuracy and stability checks using the R package *bootnet* version 1.2.4 [23]*.* We assessed the accuracy of the network connections, the stability of strength centrality, and tested whether the network connections and centrality estimates for different variables differ from each other.

## Results

### Patient characteristics

The study sample consisted of 6616 respondents, of which 49.8% were male and 50.2% female. The demographic profile distribution of subjects is reported in Table 2, and the item frequency and domain distribution are reported in sTable 1 in the Supplement. After treating “don’t know” and “refused answers” as missing data, there were overall less than 0.5% missing data on the general psychopathology, psychosis, OCD, gambling, and chronic conditions variables. In addition, there were 1.13% missing data on the variable measuring age of first alcoholic drink, and 15.45% missing data on the EQ-5D, due to the instrument being administered at a later time point than the rest of the measures. Given that the estimation methodology employed requires full data, we imputed missing data^1^ using the *mice R*-package version 3.6.0. prior to fitting the model [24].

**Table 2:** Demographic profile distribution

Demographic	Value	Frequency	%
Gender	Male	3295	49,8%
	Female	3321	50,2%
Age group	18–34	2292	34,6%
	35–49	2359	35,7%
	50–64	1551	23,4%
	65+	414	6,3%
Marital Status	Married	4293	64,9%
	Separated	31	0,5%
	Divorced	230	3,5%
	Widowed	236	3,5%
	Never Married	1826	27,6%
Language	English	5262	79,6%
	Chinese	540	8,2%
	Malay	814	12,3%

### Network analysis

**Fig. 1:**
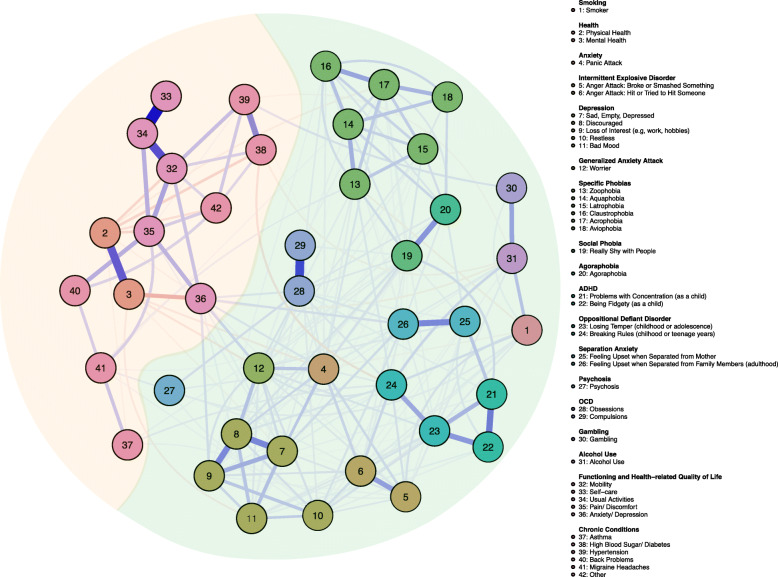
Network structure depicting the different domains of psychopathology, functioning, and chronic conditions, differentiated by colors. Blue edges indicate positive associations, red edges indicate negative associations, and the thickness of an edge represents the strength of the association

The resulting network structure is presented in Fig. 1. The *physical* and *mental health* self-report variables were not reverse-coded, a higher value thus indicating better health reports. For all other variables, a higher value indicates more problems. Overall, all nodes were associated with at least one other node in the network.

On a global level, the network was visually divided into two noticeable domains: a mental health domain consisting mainly of psychopathological nodes (to the right), and a physical health domain consisting mainly of nodes pertaining to physical problems, such as chronic conditions and functioning (to the left). We coin this conspicuously separated structure a *Cartesian graph*, after the dualist philosopher Descartes. Noticeably, however, the borders between these two domains are fuzzy and bridged by various cross-domain associations.

To summarize the results of our analysis, we will first highlight within-domain associations, followed by between-domain associations. Of note, within each domain (i.e., psychopathological and physical), there are multiple clusters differentiated by color, pre-defined according to classic diagnostic categories (such as the Diagnostic and Statistical Manual of Mental Disorders [25]). We will address these as *clusters*, to differentiate them from what we refer to as *domains* (i.e., the Cartesian graph).

#### Within-domain associations

Within-domain associations were common and stronger than between-domain associations, with most items being associated with a multitude of other items within the same domain.

Especially, the psychopathological domain displayed high connectivity, almost all associations being positive (i.e., an increase in one item predicts an increase in another item). The items belonging to attention deficit hyperactivity disorder (ADHD), oppositional defiant disorder (ODD), and separation anxiety were strongly interrelated. *Smoking*, *gambling*, and *alcohol use* were all linked with each other. The anxiety items were associated with depression and intermittent explosive disorder. *Worrier* was connected with all depression items and *panic attack*, which was in turn associated with feeling *sad, empty, depressed*, and both *anger attack* items. *Obsessions* were associated with *agoraphobia*, being *really shy with people*, and with *psychosis*.

The physical health domain, while less well-connected, displayed strong connectivity within and between functioning items and chronic conditions. Notably, chronic conditions displayed a less clear clustering pattern and did not group as well together, but were divided by the functioning items. *Mobility* was associated with *hypertension*, *high blood sugar/ diabetes*, and *other chronic conditions*. *Pain/ discomfort* was associated with *back problems* and *other chronic conditions*. Self-report measures of physical and mental health clustered within the functioning cluster, with higher reports of *physical health* being negatively associated with *mobility*, *high blood sugar/ diabetes*, *other chronic conditions* and *pain/ discomfort*. Higher reports of mental health were negatively associated especially with *anxiety/ depression*.

#### Between-domain associations

While the two domains of the Cartesian graph are prominent and less connected, the borders between these are fuzzy and bridged by various cross-domain associations. The item measuring *anxiety/ depression* according to the functioning measure was the main item connecting the two domains, located in the center of the network. This was strongly associated especially to the psychopathology items *panic attack*, *loss of interest*, *worrier*, *obsessions*, *feeling upset when separated from family members (adulthood)* and to the physical health items *mobility*, *pain /discomfort*, and *mental health* reports.

Other between-domain associations include *hypertension*, visibly and strongly associated with *gambling*, *asthma* associated with the mood item *restless*, *social phobia*, and with the ODD item *breaking rules during childhood or teenage years*. The item *migraine headaches* was associated with *psychosis*, and very weakly with the mood item *restless*, as well as with some types of *phobia*. *High blood sugar/ diabetes* was mostly, albeit weakly, negatively associated with ODD and *alcohol use.*

### Centrality analysis

**Fig. 2:**
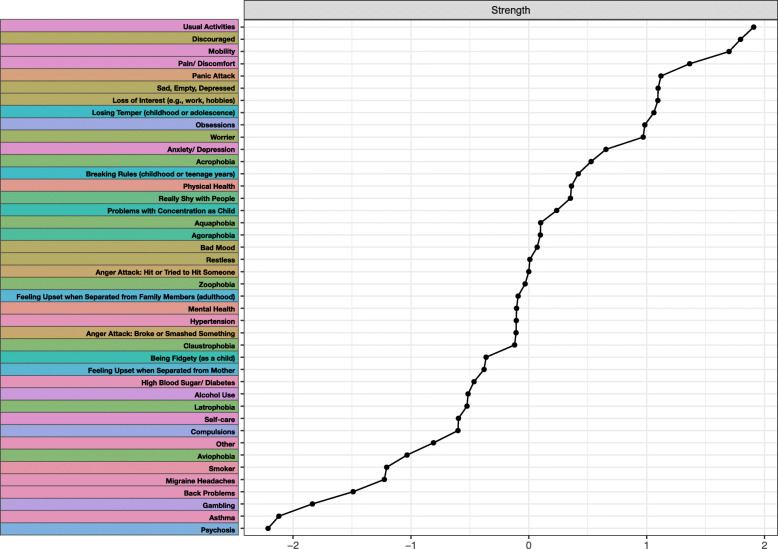
Centrality plot depicting the strength of each node in the network structure, ordered from the node with the highest strength to the node with the lowest strength in the network. Node strength quantifies how strongly a node is directly connected to other nodes in the network (i.e., by summing all absolute edge weights of edges connected to the given node). All values are standardized and higher values indicate greater centrality in the network

The centrality plot is presented in Fig. 2. The top 3 items with the highest strength centrality were *usual activities*, *discouraged*, and *mobility,*^2^ while the 3 least central items in terms of strength were *psychosis*, *asthma*, and *gambling.*^3^ sFigure 4 in the Supplement provides an overview of all the significant and non-significant differences between centrality items.

### Network Replicability and robustness

Appendix 3 and sFigures 2, 3, 4, 5 in the Supplement detail on the results of the accuracy and stability checks. Overall, our results suggest that the network model is very stable, many of the identified edges and centrality measures are significantly different from each other, and all findings are interpretable.

## Discussion

The current study used a network approach in an aim to uncover associations, at a subclinical level, between a wide array of psychopathological conditions, chronic illness and functioning. To our knowledge, this is the first study to focus on such a multitude of complex relations between different physical and health-related domains. Overall, we identified what we have labeled a *Cartesian graph*: a network graph split into two visible domains: a (mainly) psychopathological domain (more generally referred to as the mental health domain), and a (mainly) functioning and chronic conditions domain (more generally referred to as the physical health domain). The borders between these two domains are fuzzy and bridged by various cross-domain associations.

To date, there is wide evidence supporting the comorbidity between physical conditions and mental disorders [3, 4], with a majority of findings indicating mood and anxiety disorders as the main comorbid feature [26–29]. Although the current study identified few links between specific anxiety- and depression-related symptoms and chronic conditions, most chronic conditions were associated with items related to functioning, which were in turn associated to reports of anxiety/ depression–the main bridging item between the domains. Notably, the anxiety/ depression item, as well as the remaining functioning items were designed to measure the presence of *current* symptomatology, while the rest of psychopathological items were designed to measure *lifetime presence* of symptomatology. Taken together and in line with high rates of relapse for depression [30] and generalized anxiety disorders [31, 32], these findings suggest that overall lifetime symptomatology may predict current symptomatology (i.e., subjects with lifetime symptoms may report more current symptoms and vice versa), and current symptomatology may in turn be linked to current levels of functioning. Further, our results indicate that functioning plays a unique role and is a crucial bridging component in linking chronic conditions to psychopathology. It may thus be that when chronic conditions are associated with a decrease in functioning and thus low HRQoL reports, psychopathological symptoms may be triggered. Similarly, chronic psychopathology affecting daily-life functioning may lead to a rise in other physical chronic conditions. Previous research indeed identified that better functional status and fewer depressive symptoms were significantly associated with a higher quality of life in adults with chronic conditions [33]. Centrality analyses further support these findings, with functioning and depression items being most central in the current network structure. In addition, in line with outcomes showing high comorbidity between physical conditions and mental conditions [3, 4], we found that self-reports of physical and mental well-being were strongly linked together, indicating that subjects reporting poorer mental health are more likely to also report poorer physical health and vice versa.

Other between-domain links included associations between asthma and depression, social phobia, and ODD. Previous research identified that children diagnosed with and taking medication for asthma were more likely to endorse common behavioral problems [34], while lifetime and current asthma diagnosis were associated with a range of mental disorders, including social phobia and affective disorders [35]. We further found hypertension and gambling to be linked, even when controlling for alcohol use and smoking, supporting findings on the detrimental effect of gambling on physical health [36]. Further, within the psychopathology domain, smoking, gambling, and alcohol were well-clustered items, the comorbidity between the addictions being well-documented [37–39]. Smoking was further associated with psychosis, in line with evidence that smoking is common in psychotic disorders [40]. In addition, interestingly, the psychosis item was the only psychopathological item that fell in between the two domains of the network, being connected to psychopathology, but also to the chronic conditions through its association with migraine headaches. Side-effects of antipsychotic medication can include headaches [41, 42], but some evidence suggests severe forms of migraine–such as migraine aura–can also be associated with psychotic manifestation [43–45]. Psychosis and obsessions were also interrelated, indicating this association may already present at subclinical levels of psychopathology, and not only in patients [46], or in subjects at ultra-high risk for psychosis [47]. Finally, the obsessions item was one of the more central items in the network, being extensively associated to psychopathology. Of note, recent research showed OCD to have one of the largest treatment gaps (89.8%) in Singapore [48], highlighting the importance of addressing symptomatology early and encouraging help-seeking behavior.

Finally, within-domain and within-cluster associations were stronger and predominantly positive, suggesting activation may spread faster within the same domain. In addition, some psychopathology symptom clusters displayed lower connectivity to others (e.g., specific phobias) than other symptom clusters (e.g., depression, anxiety, childhood disorders), indicating the latter may be more comorbid. These results align with previous research [7] investigating the network structure of the Diagnostic and Statistical Manual for Mental Disorders (DSM) [49]. Of note, previous research [7] relied on a skip structure, which is problematic when constructing network structures [50]. The current study overcame this limitation^4^ and is thus the first to approximately assess the structure of a wide variety of mental disorder symptoms, overcoming an important limitation of earlier work.

The current research aimed to take a first step towards identifying important features in the development of the comorbidity between mental and physical health, by zooming into and bringing together a multitude of health-related domains. While the research is exploratory in nature and preliminary, a key finding of our research is the crucial role played by functioning in bridging chronic conditions and psychopathology. This finding indicates that when chronic conditions are associated with a decrease in functioning, psychopathological symptoms may be triggered and vice-versa. Functioning may thus be a potential key target for treatment: by tackling problems in functioning early on we may be able to circumvent problems arising in other health-related domains. Further, functioning was especially related to current complaints of anxiety and depression, which were in turn related to long-term psychopathological complains, adding to the importance of addressing functioning complaints in intervention strategies. In addition, we identified gambling to be one of the addictions that paved ways to both physical and mental health problems and psychosis to be the main psychopathological domain to fall in between the physical and mental health domains. These results indicate that approaching these conditions holistically by taking into account both physical and mental health complaints is essential, as leaving out any one component may lead to a faster activation of problems in that specific domain, ultimately leading to feedback loops and complaints in both physical and mental health domains. Alongside these main findings, we discussed within-domain and within-cluster associations, pinpointing to depression, anxiety, and childhood disorders as being more connected clusters and thus more likely to lead to activation of other disorders and therefore comorbidity.

Of note, as highlighted above, our study is exploratory in nature and preliminary. Future research is essential for expanding on our findings, by including more diverse samples (e.g., focus on a world-wide population, clinical populations, and so forth), as well as a wider array of variables concerned with chronic conditions. Here, due to the nature of data collected, we were limited to investigating only five types of common chronic conditions, as well as five functioning problems. Network studies designed specifically to investigate this comorbidity could expand on the inclusion and selection of variables, as to provide further information on this comorbidity. Alongside the replication of our results, this will enable better pinpointing of treatment targets, which may provide to be essential in reducing the comorbidity between mental and physical health. Ultimately, experimental designs built upon results from exploratory research can further lead to insights into treatment development.

In sum, we highlighted complex associations between a multitude of health-related domains. Our main findings include the identification of (1) a Cartesian graph consisting of a mental and a physical health domain, (2) functioning playing a crucial role in bridging chronic conditions and psychopathology, and (3) several within- and between-domain associations informative for potential pathways to comorbidity.

### Limitations

Our results should be considered in light of several limitations. First, the current study was based on cross-sectional data which precluded strong inferences on causal direction, and therefore any conclusions regarding direction of causality are tentative. Second, the WMH-CIDI [13] interview encompasses self-report statements, and may be prone to bias due to social desirability or under-reporting of symptomatology. Further, the current study focused on complete data cases and did not include severity of items in the analyses. Clinical samples may display different patterns of associations and current results were discussed in light of subclinical level of psychopathology. Finally, the study was carried out in a very specific population of residents of Singapore, and therefore the extent to which they generalize to other cultures is not yet known.

## Conclusions

This study provides rich information on the complex associations between mental health and chronic conditions. Our results highlight the central role of functioning in bridging psychopathology to chronic conditions, as well as a multitude of potential within- and between-domain pathways to comorbidity, which can often be overlooked or simplified by reductionist approaches to psychopathology. We assert investigating such unique associations between different health domains may highlight potential pathways to comorbidity, ultimately aiding research and treatment targets.

## Supplementary Information

**Additional file 1 **This *Supplementary Materials* file has been provided by the authors to give readers additional information about their work. It includes a further description of the measures used in the study, information about item frequency and domain distribution, as well as accuracy and stability checks of the network model.

## Data Availability

Data availability: Due to the strict regulations and its sensitive nature, supporting data cannot be made openly available. The data is available on request from the senior author.

## Abbreviations

HRQoL: Health-related quality of life
SMHS: Singapore Mental Health Study
WMH-CIDI: World Health Organization-Composite International Diagnostic Interview
SOGS: South Oaks Gambling Screen
ADHD: Attention deficit hyperactivity disorder
ODD: Oppositional defiant disorder
DSM: Diagnostic and Statistical Manual for Mental Disorders

## Footnotes

1 We chose imputation over listwise deletion, as most missing data were on the EQ-5D variable and were missing at random, as a result of later administration of the test. Of note, listwise deletion would have resulted in a large loss of information of data available on the other 38 variables.

2 Based on bootstrapping [23], these items were significantly different from many of the other items in the network, though not significantly different from each other and from other high centrality items, including *depressed*, *loss of interest*, and *pain/ discomfort*.

3 Based on bootstrapping [23], these items were significantly less central from many of the other items in the network, but not significantly different from each other and from other low centrality items, including *back problems* and *migraine headaches.*

4 While the design of the current study originally relied on a skip-structure (to unburden data collection), here we focused on complete cases only and did not include severity of items in the analyses (i.e., focused on sub-clinical levels of psychopathology), thus overcoming this limitation.
