# Supplementary material for: Extended network analysis: from psychopathology to chronic illness
Source: BMC Psychiatry. 2021 Feb 27;21:119. doi: 10.1186/s12888-021-03128-y (PMC7913444; doi:10.1186/s12888-021-03128-y)
Supplement: Supplementary file 1 — Additional file 1 This Supplementary Materials file has been provided by the authors to give readers additional information about their work. It includes a further description of the measures used in the study, information about item frequency and domain distribution, as well as accuracy and stability checks of the network model. [file 12888_2021_3128_MOESM1_ESM.pdf]

Supplementary Online Content

Isvoranu, A.M., Abdin, E., Chong, S. A., Vaingankar, J., Borsboom, D., Subramaniam, M.

Extended Network Analysis: From Psychopathology to Chronic Illness

Appendix 1. Measures

Appendix 1.1. WMH-CIDI Interview

Appendix 1.2. Other measures

Appendix 2. Clinical characteristics

sTable 1. Item frequency and domain distribution

Appendix 3. Accuracy and stability checks

sFigure 1. Bootstrapped 95% confidence intervals of edge weights, based on 1,000 nonparametric bootstrap samples

sFigure 2. Edge difference test, based on 1,000 nonparametric bootstrap samples

sFigure 3. Node strength stability, based on 1,000 case-drop bootstraps

sFigure 4. Centrality difference test, based on 1,000 nonparametric bootstrap samples

This supplementary material has been provided by the authors to give readers additional information about their work.

Appendix 1. Measures

We included several measures assessing psychopathology, alcohol use, gambling, major chronic conditions, and health-related quality of life. Below we include a detailed description of each measure used.

Appendix 1.1. WMH-CIDI Interview

The World Health Organization-Composite International Diagnostic Interview (WMH-CIDI)[1] was used to assess overall psychopathology. Due to the skip-structure of the WMH-CIDI, we selected and included only items that were answered by the full sample and no follow-up or disorder-specific questions.

First, we included 26 items from the *Screening Section* of the WMH-CIDI, focused on the presence of general psychopathology symptoms across lifetime. These were as follows: 1) smoking, 2) physical health self-rating, 3) mental health self-rating, 4) panic attacks, 5) anger attacks (i.e., breaking or smashing something), 6) anger attacks (i.e., hitting or attempting to hit someone), 7) depression, 8) feeling discouraged, 9) loss of interest, 10) restlessness, 11) bad mood, 12) worrying, 13) zoophobia, 14) aquaphobia, 15) latrophobia, 16) claustrophobia, 17) acrophobia, 18) aviophobia, 19) being really shy with people, 20) agoraphobia, 21) problems with concentration as a child, 22) being fidgety as a child, 23) losing temper as a child or teenager, 24) breaking rules as a child or teenager, 25) feeling upset when separated from mother, 26) feeling upset when separated from family members in adulthood. The smoking item, as well as the self-rating items related to physical and mental health were binarized due to restrictions of the model used (i.e., being a smoker was encoded as a 1, while ex-smokers or subjects who never smoked were encoded as a 0; excellent and very good mental or physical health were encoded as a 1, while good, fair, or poor were encoded as a 0).

Second, we included 1 item measuring psychosis, which was constructed based on the items measured in the *Psychosis Screen* section of the WMH-CIDI. The psychosis item was constructed as follows: If a subject endorsed any of the 6 screening items measuring psychosis (i.e., seeing a vision; hearing voices; experiencing mind control; feeling that their mind was being taken over by strange forces; experiencing attempts at communication from strange forces; believing that there was a plot to harm them), a 1 was encoded on the psychosis item included in the network. Of note, if the subject answered on the follow-up question measuring whether any of the items were endorsed when dreaming, half-asleep or under the influence of alcohol or drugs, then the original psychosis measure was endorsed as not present.

Third, we included 2 items – obsessions and compulsions – measuring obsessive-compulsive disorder, which were constructed based on the items measured in the *Obsessive-Compulsive Disorder Section* of the WMH-CIDI. The obsession item was constructed as follows: If a subject endorsed any of the 5 screening items measuring obsessions (i.e., concerns about germs or contamination; concerns about causing harm; concerns about symmetry and order; concerns about saving things; [and some other] recurrent disturbing thoughts), a 1 was encoded on the obsession item included in the network. The compulsions item was constructed in a similar manner: If a subject endorsed any of the 5 screening items measuring compulsions (i.e., repeatedly washing, cleaning, or decontaminating; checking things; ordering or touching things; saving things; [and some other] behaviors they did over and over), a 1 was encoded on the compulsions item included in the network.

Fourth, we included the first item of the *Alcohol Use* section of the WMH-CIDI, measuring the age of drinking the first alcoholic beverage. If a subject reported that they never drank alcohol, this was encoded as a 0 on the item measuring alcohol use. All answers

providing the age of the first alcoholic beverage, suggesting that the subject drinks alcohol, were encoded as a 1.

Appendix 1.2. Other measures

We included several other measures, assessing gambling, major chronic conditions, and health-related quality of life.

First, we included 1 item measuring gambling, which was constructed based on the South Oaks Gambling Screen (SOGS)[2]. The gambling item was constructed as follows: If a subject endorsed any of the 11 screening items measuring gambling (i.e., playing cards for money; betting on horses, dogs, or other animals; betting on sports; paying dice games, including craps, over and under or other dice games; going to casinos; playing the numbers or betting on lotteries; playing bingo; playing stock and/ or commodities market; playing slot machines, poker machines, or other gambling machines; bowling, shooting pool, paying golf, or some other game of skill for money; playing pull tabs or "paper" games other than lotteries), for more than once a week, a 1 was encoded on the gambling item included in the network.

Second, we included 6 items measuring the presence chronic conditions, based on the modified version of the CIDI checklist of chronic medical conditions. We included as standalone items the items with above 5% endorsement (i.e., asthma; high blood sugar/ diabetes; hypertension; back problems; migraine headaches), to allow for some variability in the data, as restricted by the model used. All items below that had under 5% endorsement were encoded in the "Other" category item as follows: If a subject endorsed any of the 10 items (i.e., arthritis or rheumatism; cancer diagnosed within the last 3 years; a neurological condition, such as epilepsy, convulsions, fainting spells, or Parkinson's disease; stroke or major paralysis; a heart attack, coronary heart disease, angina, congestive heart failure or

other heart disease; stomach ulcer; choric inflamed bowel, enteritis, or colitis; thyroid disease; kidney failure; chronic lung disease such as bronchitis or emphysema), a 1 was encoded on the other chronic conditions item included in the network.

Third, we included 5 items measuring quality-of life and functioning, based on the EQ-5D[3]. These were as follows: mobility, self-care, usual activities, pain/ discomfort, and anxiety/ depression. Due to restrictions of the model, the items were binarized, with no problems being encoded as a 0, and some problems/ unable to do encoded as 1.

Appendix 2. Clinical characteristics

sTable 1. Item frequency and domain distribution

Item	Domain	Frequency	%
Smoking	CIDI Screening Section	2062	31,2%
Physical Health*	CIDI Screening Section	5329	80,5%
Mental Health*	CIDI Screening Section	6059	91,6%
Panic Attack	CIDI Screening Section	1995	30,2%
Anger	CIDI Screening Section	1004	15,2%
Lost Control	CIDI Screening Section	893	13,5%
Depressed	CIDI Screening Section	1797	27,2%
Discouraged	CIDI Screening Section	1669	25,2%
Loss Interest	CIDI Screening Section	1286	19,4%
Restless	CIDI Screening Section	425	6,4%
Bad Mood	CIDI Screening Section	869	13,1%
Worrier	CIDI Screening Section	1681	25,4%
Zoophobia	CIDI Screening Section	1886	28,5%
Aquaphobia	CIDI Screening Section	873	13,2%
Latrophobia	CIDI Screening Section	1002	15,1%
Claustrophobia	CIDI Screening Section	517	7,8%
Acrophobia	CIDI Screening Section	1060	16,0%
Aerophobia	CIDI Screening Section	317	4,8%
Very Shy	CIDI Screening Section	993	15,0%
Agoraphobia	CIDI Screening Section	501	7,6%

Concentration Problems Child	CIDI Screening Section	350	5,3%
Restless Child	CIDI Screening Section	229	3,5%
Trouble Child	CIDI Screening Section	532	8,0%
Break Rules Child	CIDI Screening Section	1896	28,7%
Separation Child	CIDI Screening Section	563	8,5%
Separation	CIDI Screening Section	591	8,9%
Psychosis	CIDI Psychosis Screen	306	4,6%
Obsessions	CIDI Obsessive-Compulsive Disorder Section	581	8,8%
Compulsions	CIDI Obsessive-Compulsive Disorder Section	388	5,9%
Gambling	South Oaks Gambling Screen (SOGS)	810	12,2%
Alcohol Use	CIDI Alcohol Use Section	3358	50,8%
Mobility	EQ-5D	225	3,4%
Self-Care	EQ-5D	34	0,5%
Usual Activities	EQ-5D	143	2,2%
Pain/ Discomfort	EQ-5D	840	12,7%
Anxiety/ Depression	EQ-5D	488	7,4%
Asthma	CIDI checklist of chronic medical conditions	685	10,3%
High Blood Sugar/ Diabetes	CIDI checklist of chronic medical conditions	653	9,9%

Hypertension	CIDI checklist of chronic medical conditions	1095	16,5%
Back Problems	CIDI checklist of chronic medical conditions	436	6,6%
Migraine Headaches	CIDI checklist of chronic medical conditions	446	6,7%
Other	CIDI checklist of chronic medical conditions	1179	17,8%

*Denotes reverse-coded items (i.e., a higher value indicates better health)

Appendix 3. Accuracy and stability check for the estimated network model

In order to check whether the estimated network connections and centrality measures were accurate, we carried out bootstrap stability checks, using the R-package *bootnet,* version 1.2.4[4]. sFigure 1 below displays bootstrap results for all edge weights, while sFigure 2 displays the average case-drop bootstraps for strength centrality. In addition, sFigure 3 and sFigure 4 below displays the difference test results for the network connections and centrality estimates for different variables. An extensive interpretation of each result is included in the respective figure captions.

sFigure 1. Bootstrapped 95% confidence intervals of edge weights, based on 1,000 nonparametric bootstrap samples

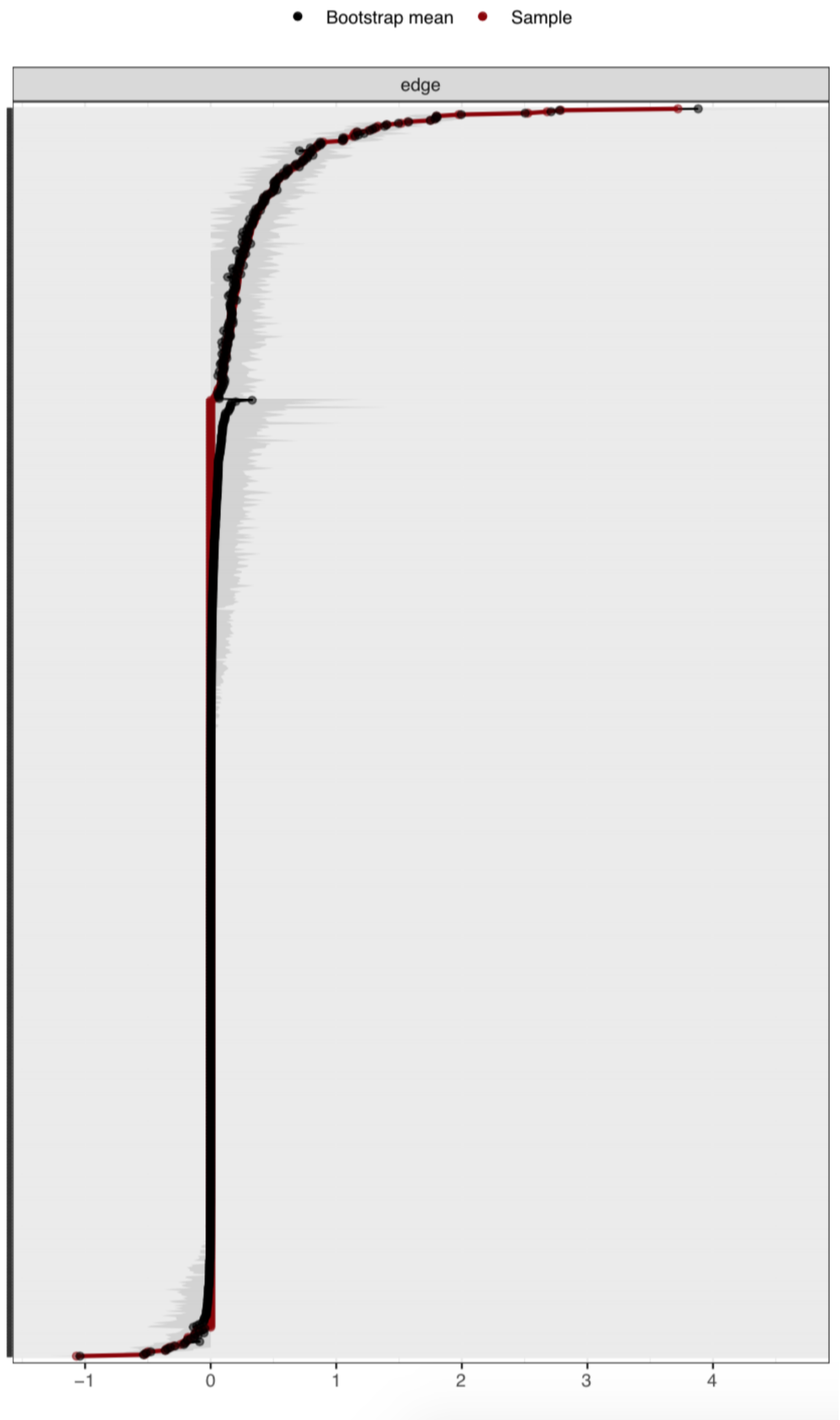

To investigate the stability of edge weight parameters, we utilized nonparametric bootstrap methods, and constructed a 95% bootstrapped confidence interval around the regularized

edge weight. When bootstrapping, a new dataset is computed through re-sampling (with replacement) of the original data (here 1,000 iterations), thus giving insight into the edge weight variations across different bootstraps. **sFigure 1** displays these results: the red dots represent the original sample values, the black dots represent the bootstrap means, and the grey areas represent the 95% bootstrapped confidence interval. A narrow interval indicates that the stability is very good, while a wider interval indicates high variability and thus lower stability. Here the bootstrapped confidence interval is very narrow, suggesting highly stable and interpretable results.

sFigure 2. Edge difference test, based on 1,000 nonparametric bootstrap samples

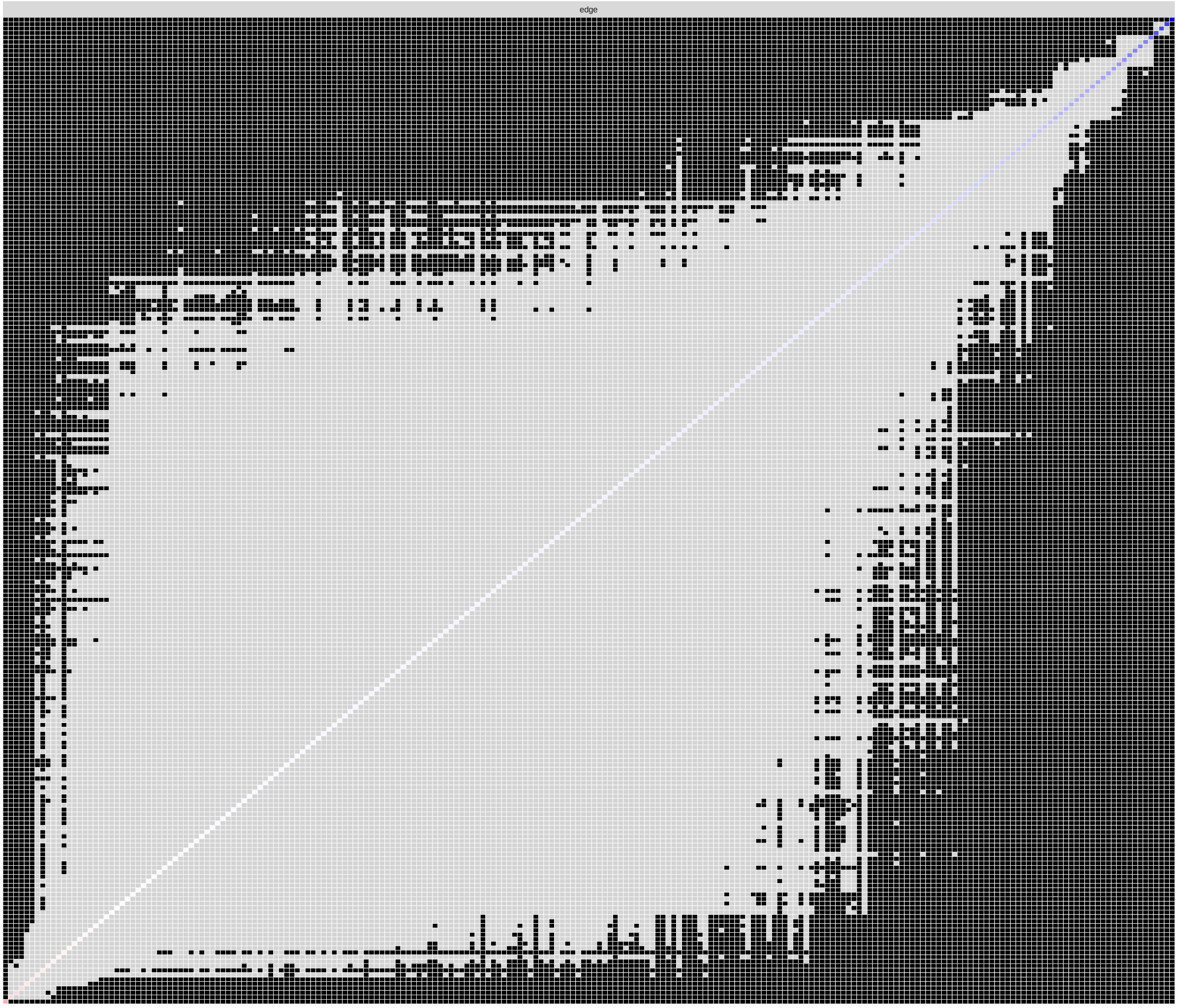

To investigate whether the edges identified in the network are significantly different than each other in terms of strength, we carried out a bootstrap stability difference test (α = 0.05). Each point on the x and y axes represents a pair of edges identified in the network; of note, here the labels are omitted for clarity. The gray boxes indicate that two edges do not significantly differ from each other, while the black boxes indicate that two edges significantly differ from each other. The diagonal represents the edge strength, and the edges are order from weaker edges to stronger edges (i.e., from white to dark blue). Many edges here, especially the stronger edges, are significantly different than other edges, thus indicating that generally edge strength comparison is adequate.

sFigure 3. Node strength stability, based on 1,000 case-drop bootstraps

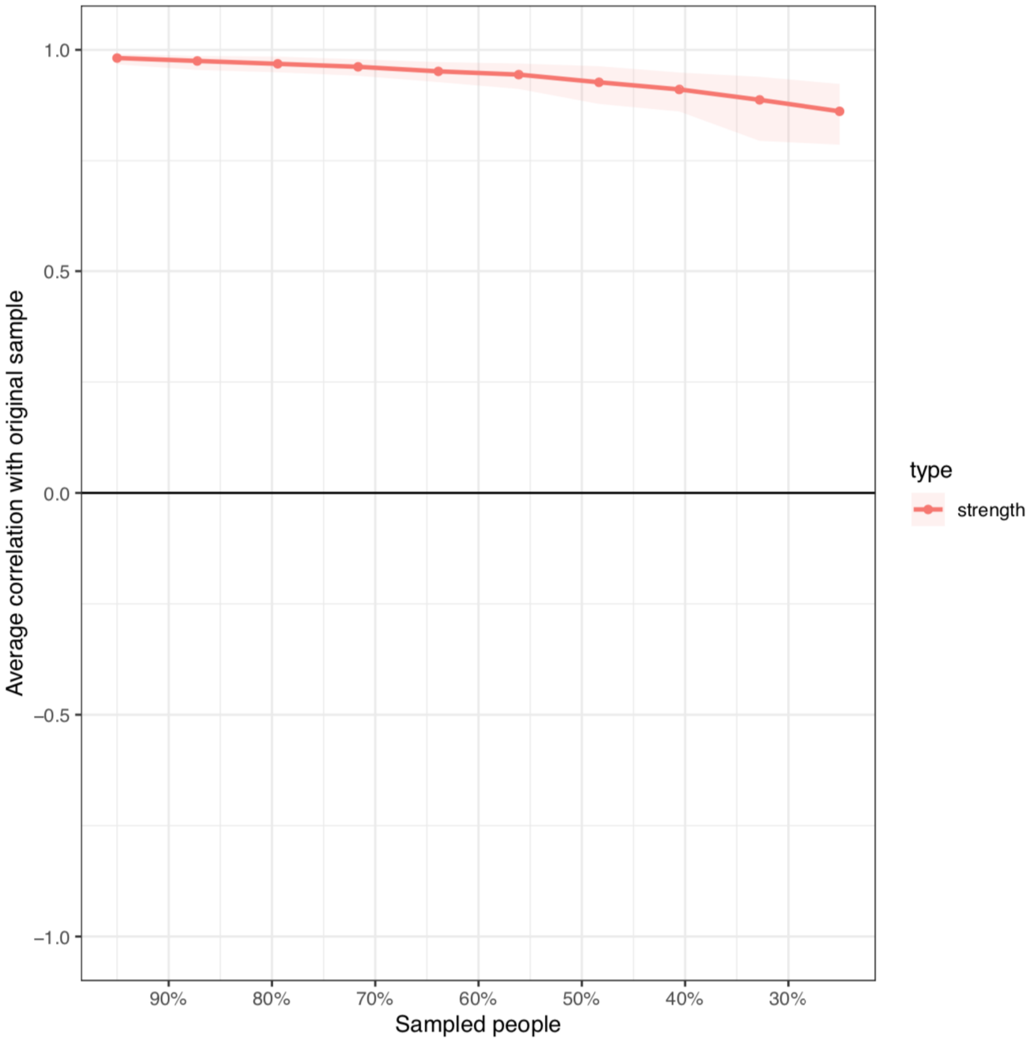

To investigate the stability of the strength centrality indices, we used a case-dropping bootstrap procedure (i.e., re-estimated the centrality indices with an increasingly higher percentage of dropped-out cases from data). We then estimated the correlation between the original centrality indices and those obtained from the reduced subsamples. **sFigure 3** above presents these results. To quantify the findings, we further computed the correlation stability coefficient (CS-coefficient), representing the maximum proportion of cases which can be dropped from the study population to maintain a correlation of at least .70 with the original centrality indices. The recommendation of Epskamp and colleagues[4] is that the CS-coefficient should be minimum .25, but ideally higher than .50. The CS-coefficient here was 0.75, thus indicating reliable results.

sFigure 4. Centrality difference test, based on 1,000 nonparametric bootstrap samples

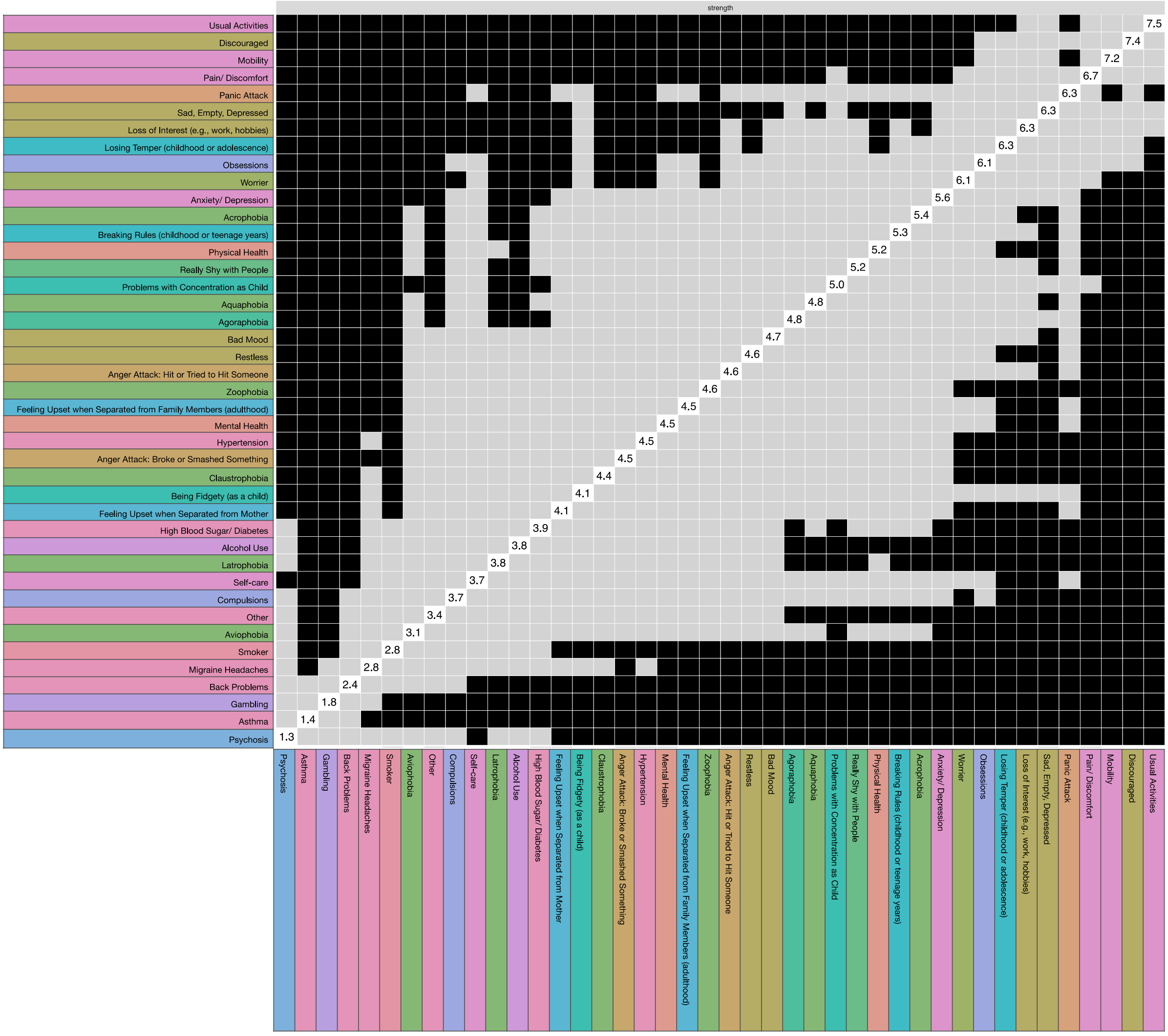

To investigate whether the nodes in the network were significantly different than each other in terms of strength centrality, we carried out a bootstrap stability difference test (α = 0.05). Each point on the x and y axes represents a node in the network. The gray boxes indicate that two nodes do not significantly differ from each other, while the black boxes indicate that two nodes significantly differ from each other. The numbers in the diagonal represent the values of the strength centrality measure of the specific node. The most and least central nodes in the network are generally identified as significantly different than most of the other nodes, though not significantly different from each other.

References

1. Kessler RC, Üstün BB. The World Mental Health (WMH) Survey Initiative version of the World Health Organization (WHO) Composite International Diagnostic Interview (CIDI). *Int J Methods Psychiatr Res*. 2004. doi:10.1002/mpr.168
2. Lesieur HR, Blume SB. The South Oaks Gambling Screen (SOGS): A new instrument for the identification of Pathological gamblers. *Am J Psychiatry*. 1987. doi:10.1176/ajp.144.9.1184
3. Herdman M, Gudex C, Lloyd A, et al. Development and preliminary testing of the new five-level version of EQ-5D (EQ-5D-5L). *Qual Life Res*. 2011. doi:10.1007/s11136-011-9903-x
4. Epskamp S, Borsboom D, Fried EI. Estimating psychological networks and their accuracy: A tutorial paper. *Behavior Research Methods*. 2017:1-18.
